# Calcium imaging, MEA recordings, and immunostaining images dataset of neuron-astrocyte networks in culture under the effect of norepinephrine

**DOI:** 10.1093/gigascience/giy161

**Published:** 2018-12-13

**Authors:** Yasmin Bar El, Sivan Kanner, Ari Barzilai, Yael Hanein

**Affiliations:** 1 School of Physics and Astronomy, Tel-Aviv University, 30 Chaim Levanon street, Tel- Aviv, 6997801, Israel; 2 Department of Neurobiology, Faculty of Life Sciences, Tel-Aviv University, 30 Chaim Levanon street , Tel-Aviv, 6997801, Israel; 3 Sagol School of Neuroscience, Tel-Aviv University, 30 Chaim Levanon street, Tel-Aviv, 6997801, Israel; 4 School of Electrical Engineering, Tel-Aviv University, 30 Chaim Levanon street, Tel-Aviv, 6997801, Israel

**Keywords:** astrocytes, norepinephrine, MEA, calcium imaging, neuron-astrocyte communication

## Abstract

**Background:**

Monitoring the activity and morphology of neuron-astrocyte networks in culture is a powerful tool for studying dynamics, structure, and communication in neuron-astrocyte networks independently or as a model of the sub-brain network. These cultures are known to produce stereotypical patterns of activity, e.g., highly synchronized network bursts resembling sleep or seizure states, thus it enables the exploration of behaviors that can relate to brain function and disease. High-resolution microscopy of calcium imaging combined with simultaneous electrical recording provides a comprehensive overview on the network's dynamics. This setup makes it possible to apply global perturbations of electrical and chemical stimulation on the cultures during the recording task and to record the effects on network activity on-line. Morphological changes in the cultures can be obtained to have a complete dataset for structure-function study of neuron-astrocyte networks *in vitro*.

**Findings:**

The 4 TB of data presented here was recorded and imaged as part of an accompanying study looking at *in vitro* structure-function of neuron-astrocyte networks. Simultaneous optical (calcium imaging) and electrical (micro-electrode array) recordings lasted 5–12 minutes and included spontaneous activity recording, electrical and chemical stimulation of neuron-astrocyte, and isolated astrocyte cultures. The data include activity recordings of 58 different cultures, with 1–2 regions of interest recorded for each culture. Production procedures, experimental protocols, and reuse options are included. The data have been suitable to reveal changes in the activity and morphology of the cultures and enabled observation and analysis of neuron-astrocyte and isolated astrocyte culture behaviors under the applied perturbations.

**Conclusions:**

Our dataset is sufficient to show significant changes in activity and morphology of neuron-astrocyte networks in culture under the applied stimulations. More than 100 recordings of 58 different cultures give insight of the observation's significance and led to conclusions about astrocyte activity and neuron-astrocyte network communication. Making it available here will allow others to test new tools for calcium imaging analysis and extracellular neuronal voltage recordings.

## Data Description

### Context

The data presented here were generated as part of an accompanying publication studying *in vitro* structure-function of neuron-astrocyte networks [1]. The aim of the study was to explore changes in activity, morphology, and inter-network communication between astrocytes and neuronal networks in culture under the effect of norepinephrine (NE). The data contain raw recordings of neuron-astrocyte and astrocyte-only cultures as well as immunostaining images. All recorded and imaged data are of cortical cultures of mice 0 or 1 day post-natal, prepared as described in the Methods section of [1] and in more detail in the protocols provided via protocols.io [2]. Detailed documentation of the dataset structure, sorting, and naming of files and folders is provided in the readme.txt file located in the datasets main folder and below.

The following protocols were used for data production and are available::

- Primary cortical neuronal-astrocyte cell culture preparation
- Isolated astrocyte cell culture preparation
- Isolated astrocyte cell culture preparation—alternative protocol
- Immunocytochemistry for morphology analysis
- Electrophysiology recording by micro-electrode array (MEA)
- Calcium imaging recording

The dataset includes the following:

Please see the “readme.txt” file for further explanation on the file and folder naming system.

- Tif stack file: raw calcium imaging recording in tif files.
- Bmp files of immunostaining images used for morphological analysis
- Mcd files: can be viewed by MC-RACK software by Multichannel Systems (MCS) [3]. The software allows a replay of the data as seen during the experiment. The default view presents the electrodes voltage as a function of time on a map of electrodes matrix with respect to the physical positioning of the electrodes under the culture, thus allowing an easy observation of the culture voltage activity with respect to the complementary calcium imaging activity in the same field of view (FOV). The indexes of electrodes positioned in the FOV and stimulated are detailed in the table of files on the dataset web page. The software also offers a variety of analysis tools relevant for this data, e.g., spike, sorting, and raster plots. The mcd files can be viewed in Matlab using the neuroshare Matlab toolbox by MCS and can be converted into other formats, e.g., hdf5 using MCS data manager by MCS [4]. All are available for free download on MCS website.
  Each mcd file contains the following data: i, ...
  - Raw and filtered extracellular voltage recordings, recorded using MEA setup by MCS
  - Trigger times of electrical stimulation (ES) and manual pharmacology insertion during the recording
  - Time stamps of the charge-coupled device (CCD) frame acquisition times

### Methods

Cultured neuron-glia networks organize into electrically viable networks without external guidance or chemical cues, while retaining many structural and functional features of the real brain networks [5]. After cell seeding, networks of millions of neurons and glia cells are formed. Neurons grow axons and dendrites, glia cells undergo intensive division and become excitable, cells migrate, and a network with chemical synaptic connections and gap junctions is formed [6, 7].

The experimental setup was built according to previous studies in our lab [8, 9]. Neuron-astrocyte and isolated astrocyte cultures were prepared, recorded, and imaged according to the protocols described here and in the accompanying publication [1] and also provided in more detail via protocols.io [2].

Experiment recording protocol was as follows:

Each recording time span (5–12 minutes) was equally divided into different sections:

- Spontaneous activity (SA) recording.
- ES recording and application of 25–30 seconds. Each ES included two electrodes (or fewer, see Data validation section below) located in the FOV of the calcium imaging. Electrodes selection for ES and ground was managed using MEA-select software by MCS, which was connected to the MC-card purchased from MCS. ES timings, frequency, and amplitude were programed by MC stimulus software by MCS and can be viewed in the mcd file.
- Pharmacology procedure recording and application lasted approximately 30 seconds. Time stamps of pharmacology procedure (change of 1 mL recording medium by fresh medium or by 50 μM NE medium) were manually reported into the MC-RACK during the recording task.
  - Medium change (CT) for control experiment.
  - NE addition for test experiment.

The exact timings of the ES and pharmacology (CT or NE) for each experiment, patterns, and external triggers for the CCD camera were programed by MC-stimulus software by MCS and are available in the mcd file under ‘triggers’.

The order of manipulations during the experiment were as follows (applied consecutively in time from left to right): SA-ES-SA-pharmacology-SA-ES-SA. Where pharmacology is NE or CT for test or control experiment, respectively, and all abbreviations are as described above.

### Data validation and quality control

The quality of the data was ascertained through visual inspection of the calcium imaging movies (created from the recorded stacks of tif files by Matlab or ImageJ) and MEA recordings. Primary criteria were as follows:

- Calcium imaging: clear visualization, quality of calcium dye penetration and good resolution of cells fluorescence compared to the background staining, no moving of the FOV during the recording.
- MEA recording: noisy electrodes were grounded.
- MEA electrical stimulation was possible only in cultures in which there were cells located on top of the electrodes area (cultures rarely developed outside the electrode region). This condition was observed during the experiment by bright field light and by the occurence of extracellular voltage signal read by the electrodes, before starting the recording. In these cases, the FOV was chosen such that it will include the stimulating electrodes in order to inspect the optical calcium response of the culture to the electrical stimulation. In the few cases where there were no calls around the electrode area, the experiment did not include ES (only chemical stimulation: SA-pharmacology-SA) and the FOV was chosen only by the goodness of fluorescence of the cells (condition number 1).

Recordings containing severe artifacts were discarded, an example of negative data can be found in the GigaDB entry in the folder “NegativeData” as explained below in the Availability of supporting data section. A total of 107 representative recordings have been deposited in GigaDB [10].

The analysis of this dataset was peer reviewed by the reviewers of the accompanying publication in PLoS One [1].

### Reuse potential

Our data of culture activity recordings can be further analyzed to find different aspects of dynamic signaling of neuron-astrocytes cultures *in vitro* and of NE influence on such cultures. Pharmacological treatment, ES, and spontaneous activity recordings of culture grown on MEA can be a measure of various aspects of culture dynamics, as reviewed in [11], information transfer [12], and functional plasticity [13]. Calcium imaging data can be used for quantification of calcium dynamics in the cultures [14], e.g., single-cell calcium activity and extracellular calcium waves in the astrocytes network. In addition, our data can be used as supportive data for theoretical investigation such as network dynamics modeling and computer simulations [9, 15–20]. Finally, it can be used as test data for examination of new tools for calcium imaging and extracellular neuronal voltage recordings analysis, from the level of image and signal processing of raw data to the study of network behaviors by tools for spike and burst detection, firing frequencies, and inter-burst intervals calculations [12, 14, 21–24].

The immunostaining images can be used for different morphological analysis. There is inherent value in the primary cultured neuron-astrocyte dataset due to the inclusion of a high number of co-cultures in this study. This dataset describes the response of 58 different neuron-astrocyte cultures from 11 different preparations and explores calcium signaling and morphological change in this cell culture model. The morphological analysis outlined here explored FOVs of entangled neuron-astrocyte networks, and there is clear scope for re-use of this dataset as a means of studying cell morphological features of astrocytes, e.g., astrocytes processes structure, within the neuron-astrocyte networks. Moreover, it can be used to explore different methods of astrocytes morphology evaluation of entangled cultures or new image processing tools [25]. In addition, from both a morphological and calcium dynamic perspective, the image data could be re-used as a baseline training set for computational analyses that seek to explore these phenomena in more complex neuronal-glial 2D organoid systems.

## Availability of supporting data

The dataset supporting the results presented here is available in the *GigaScience* Database online repository [10]. A list of all files and directories is available in the dataset table presented with the dataset (under Additional details) and available for download from the ftp. The data structure and major directories in which it was divided to are described below. The main folders in the dataset are:

April2016, April2017, August2016, January2016, March2016, March2017, March31_2016, May2016, November2016, October2016, scripts and MorphologyData.

Each folder, which is named by a month and year, holds the following:

- All tif files that were recorded in the month and year of the folders name. The tif files are named as explained below and they are listed in the dataset table.
- A folder named month-year-MCS (as explained below) that holds the complementary mcd files that were recorded in the same experiments of the tif files and are numbered with respect to the tif files 3 digits as explained below.

Directories and files are named in the following structure:

“April2016/8485 × 20B2H307SunMay012016221.tif.” The files were separated into major folders under the names of month and year in which the experiments took place, e.g., the “April2016” folder contains all tif and mcd files of experiments executed in April 2016. The tif recording files were named and numbered (including date) by the Andor software used for the CCD recording [26], e.g., “8485 × 20B2H307SunMay012016221.tif.” Each CCD recording is separated into several consecutive files under the same name with an additional ending of the form filenameX#, e.g., 8485 × 20B2H307SunMay012016221 × 2. In order to relate a mcd recording to a tif stack series, we refer to the recording only by the last 3 digits of the name, i.e., “221” in this case. The complementary mcd file was named with respect to these 3 digits: April2016MCS221.mcd located in the folder: “April2016.”

The tif files are listed in the dataset table (under Additional details) with the following details:

- Culture type: “Neuron-astrocyte mixed culture” or “astrocyte culture.”
- Culture ID—consecutive numbering to distinguish the cultures from one another. Ranges from 1 to 58.
- Number of FOV in this culture; for each culture, a couple (1–2) of FOVs were recorded, also named “region of interest” in the literature.
- CT/NE—for control experiment or test experiment, respectively. In control experiment, the pharmacological treatment is change of 1 mL recording medium with fresh recording medium. For test experiment, the pharmacological treatment is an exchange of 1 mL recording medium with 1 mL 50 μL NE medium.
- Indexes of electrodes located in the recorded FOV and indexes of stimulated electrodes.

The folder “MorphologyData” holds all immunostaining images that were used for analysis of astrocytes morphology as presented in Figure 3 of the accompanying publication [1]. The bmp files are named with the following information:

**Table:** 

**Contributor Role**	**Role Definition**	
**Conceptualization**	Ideas; formulation or evolution of overarching research goals and aims.	All
**Data Curation**	Management activities to annotate (produce metadata), scrub data, and maintain research data (including software code, where it is necessary for interpreting the data itself) for initial use and later reuse.	Y.B.,S.K.
**Formal Analysis**	Application of statistical, mathematical, computational, or other formal techniques to analyze or synthesize study data.	Y.B.,S.K.
**Funding Acquisition**	Acquisition of the financial support for the project leading to this publication.	Y.H.,A.B.
**Investigation**	Conducting a research and investigation process, specifically performing the experiments, or data/evidence collection.	Y.B.,S.K.
**Methodology**	Development or design of methodology; creation of models	Y.B.,S.K.
**Project Administration**	Management and coordination responsibility for the research activity planning and execution.	Y.B.
**Resources**	Provision of study materials, reagents, materials, patients, laboratory samples, animals, instrumentation, computing resources, or other analysis tools.	Y.H.,A.B.
**Software**	Programming, software development; designing computer programs; implementation of the computer code and supporting algorithms; testing of existing code components.	Y.B.
**Supervision**	Oversight and leadership responsibility for the research activity planning and execution, including mentorship external to the core team.	Y.B.
**Validation**	Verification, whether as a part of the activity or separate, of the overall replication/reproducibility of results/experiments and other research outputs.	Y.B.
**Visualization**	Preparation, creation, and/or presentation of the published work, specifically visualization/data presentation.	Y.B.
**Writing—Original Draft Preparation**	Creation and/or presentation of the published work, specifically writing the initial draft (including substantive translation).	Y.B.
**Writing—Review and Editing**	Preparation, creation, and/or presentation of the published work by those from the original research group, specifically critical review, commentary, or revision—including pre- or post-publication stages.	All

- Control/test experiment image is named ctrl or NE, respectively.
- The exposure time to the pharmacological treatment before the culture was fixed: 1, 5, or 40 minutes (see Method section in [1]).
- The numbering of the images.

For example, “ctrl5_1” is the file name of control experiment, 5 minutes of exposure to pharmacology treatment, and it is image number 1 of this series.

The folder “scripts” contains the custom Matlab code used for the data analysis in the accompanying publication [1] to assist in reproduction. This includes MEA (mcd) and morphology (bmp) analysis. Each script has a detailed explanation of its purpose, use, and the figure it was used to build in the accompanying publication [1]. A readme file explaining how to use the code for data analysis is available in the scripts folder.

The preliminary extraction of calcium imaging data (from the tif files), i.e., extraction of individual cells fluorescence traces as a function of time, can be done using a number of open source tools available on the web for this purpose, such as ImageJ (https://imagej.net/). To automate this process, other more complex software and tool boxes are described and published, including Romano et al. [23] Mukamel et al. [26].

The folder “NegativeData” contains a representative calcium imaging recording that was discarded from analysis due to movement of the FOV during the recording. The movement can be seen starting at time 00:50.

## Abbreviations

CCD: charge-coupled device; ES: electrical stimulation; FOV: field of view; MCS: multichannel systems; CT: medium change in control experiment; MEA: micro-electrode array; NE: norepinephrine; SA: spontaneous activity.

## Ethics statement

All animal care and experiments presented in this study were conducted according to the animal research guidelines from Tel Aviv University and were approved by the Tel Aviv University Animal Care Committee (permit number –L-14-019).

## Competing interests

The authors declare that they have no competing interests.

## Funding

This work was supported by the European Research Council funding under the European Community's Seventh Framework Program (FP7/2007–2013)/ERC grant agreement FUNMANIA-306707 (to Y.H.; https://erc.europa.eu), Israel Science Foundation grants (549/12 and 41/15), German Israeli Foundation grant (I-192-418.13-2014), and Joint Italian-Israeli Laboratory on Application of Neuroscience grant (590308) (A.B.). The funders had no role in study design, data collection and analysis, decision to publish, or preparation of the manuscript.

## Supplementary Material

Response_to_Reviewer_Comments_Original_Submission.pdf

Response_to_Reviewer_Comments_Revision_1.pdf

Reviewer_1_Report_Revision_1 -- Chris Armit 9/24/2018 Reviewed

Reviewer_2_Report_Revision_1 -- Stephen Eglen 11/9/2018 Reviewed

Reviewer_3_Report_Revision_1 -- Thomas Wachtler 11/20/2018 Reviewed
