# Supplementary material for: Calcium imaging, MEA recordings, and immunostaining images dataset of neuron-astrocyte networks in culture under the effect of norepinephrine
Source: Gigascience. 2018 Dec 13;8(2):giy161. doi: 10.1093/gigascience/giy161 (PMC6351728; doi:10.1093/gigascience/giy161)
Supplement: Response_to_Reviewer_Comments_Original_Submission.pdf [file giy161_response_to_reviewer_comments_original_submission.pdf]

Author's Response To Reviewer Comments

Close

Dear Editor,
The 'Data_note_revised' has all required changes.
Thank you,
Yasmin

Close
