# Supplementary material for: Calcium imaging, MEA recordings, and immunostaining images dataset of neuron-astrocyte networks in culture under the effect of norepinephrine
Source: Gigascience. 2018 Dec 13;8(2):giy161. doi: 10.1093/gigascience/giy161 (PMC6351728; doi:10.1093/gigascience/giy161)
Supplement: Response_to_Reviewer_Comments_Revision_1.pdf [file giy161_response_to_reviewer_comments_revision_1.pdf]

Author's Response To Reviewer Comments

Close

The response file is attached as a supplemental file.

Close
