# Supplementary material for: Calcium imaging, MEA recordings, and immunostaining images dataset of neuron-astrocyte networks in culture under the effect of norepinephrine
Source: Gigascience. 2018 Dec 13;8(2):giy161. doi: 10.1093/gigascience/giy161 (PMC6351728; doi:10.1093/gigascience/giy161)
Supplement: Reviewer_1_Report_Revision_1 -- Chris Armit — 9/24/2018 Reviewed [file giy161_reviewer_1_report_revision_1.pdf]

Reviewer Report

Title: Calcium imaging, MEA recordings and Immunostaining images dataset of Neuron-Astrocyte Networks in Culture under the Effect of Norepinephrine

Version: Revision 1 **Date:** 9/24/2018

Reviewer name: Chris Armit

Reviewer Comments to Author:

This Data Note details a large volume (4TB) dataset of a co-culture neuronal-glial model that can be used to study neuron-astrocyte network dynamics and morphology. The manuscript is well-written, and there is inherent re-use potential of the primary cultured neuron-astrocyte dataset due to the high number of co-cultures included in this study. The neuron-astrocyte co-culture dataset describes the response of 58 different neuron-astrocyte cultures from 11 different preparations, and explores calcium wave propagation and morphological change in this in vitro model system.

The study outlined in this manuscript explored the effects of norepinephrine in astrocytes and neuronal networks in terms of changes in activity, morphology and inter-network communication. The data contains raw recordings of neuron-astrocyte and astrocyte-only cultures, and utilises immunocytochemistry to aid in morphological analysis. The morphological analysis outlined in this manuscript explored FOVs (field-of-views) of entangled neuron-astrocyte networks, and there is clear scope for re-use of this dataset as a means of establishing high granularity single cell morphological classifiers of neuronal and glial cells within the neuron-astrocyte networks. In addition, from both a morphological and calcium dynamic perspective, these data could be re-used as a baseline training set for computational analyses that seek to explore these phenomena in more complex neuronal-glial 3D organoid systems.

Minor comments

The authors are to be commended for making the image data available in TIF and BMP format, which are universally recognised. However, the image data are organised in directories relating to the months in which they were acquired which is not very helpful to an outside researcher who wishes to re-use this dataset. If these data are to be archived in GigaDB, then there needs to be clear descriptions for each of these files so that a researcher can unambiguously determine whether a dataset is of: a) an astrocyte culture, or; b) a neuron-astrocyte co-culture, and whether these images show; c) control treatment, or; d) norepinephrine treatment. There is inherent value in additionally including the time-point in the file descriptions.

The primary antibody incubation step outlined in protocols.io suggests that two distinct primary antibodies were incubated simultaneously (step 5 of the protocol). Likewise, in the following step of the protocol two distinct secondary antibodies were incubated simultaneously (step 6 of the protocol). The incubation of multiple antibodies simultaneously is a non-standard procedure and I invite the authors to

confirm that the published protocol is accurate, and that sufficient negative controls (i.e. omitting each primary antibody and each secondary antibody) were accomplished to ensure that cross-reactivity was not observed between the various antibody combinations, and that auto-fluorescence is not an issue. Negative controls of the immunocytochemistry data were not submitted and so I cannot determine this directly. Furthermore, I cannot see orange fluorescence - indicative of Cy3 fluorescence - in the images that I have inspected despite mention of a Cy3-conjugated secondary antibody in the protocol. The protocol in question can be found at the following link:
https://www.protocols.io/view/immunocytochemistry-tqdems6

The Micro-Electrode Array (MEA) recordings are provided in a MCD format and can be opened using the Multi-Channel Data (MCD) Manager that is made freely available by Multi Channel Systems. However, if these data were additionally made available in a HDF5 format, then it would broaden their potential for re-use as the researcher could use NumPy Python libraries or similar to run computations across these datasets. If it is straightforward to export these data in HDF5 format, I suggest that the authors additionally provide this.

The MATLAB scripts were used previously in the following publications.

* Wallach G, Lallouette J, Herzog N, De PittÃ M, Jacob E Ben, Berry H, et al. Glutamate Mediated Astrocytic Filtering of Neuronal Activity. Graham LJ, editor. PLoS Comput Biol. 2014;10: e1003964. doi:10.1371/journal.pcbi.1003964
* Herzog N, Shein-Idelson M, Hanein Y. Optical validation of in vitro extra-cellular neuronal recordings. J Neural Eng. 2011;8: 056008. doi:10.1088/1741-2560/8/5/056008

To ensure reproducibility I request that the authors additionally submit the MATLAB scripts to GigaScience / GigaDB.

Level of Interest

Please indicate how interesting you found the manuscript: Choose an item.

Quality of Written English

Please indicate the quality of language in the manuscript: Choose an item.

Declaration of Competing Interests

Please complete a declaration of competing interests, considering the following questions:

- Have you in the past five years received reimbursements, fees, funding, or salary from an organisation that may in any way gain or lose financially from the publication of this manuscript, either now or in the future?

- Do you hold any stocks or shares in an organisation that may in any way gain or lose financially from the publication of this manuscript, either now or in the future?
- Do you hold or are you currently applying for any patents relating to the content of the manuscript?
- Have you received reimbursements, fees, funding, or salary from an organization that holds or has applied for patents relating to the content of the manuscript?
- Do you have any other financial competing interests?
- Do you have any non-financial competing interests in relation to this paper?

If you can answer no to all of the above, write 'I declare that I have no competing interests' below. If your reply is yes to any, please give details below.

I declare that I have no competing interests.

I agree to the open peer review policy of the journal. I understand that my name will be included on my report to the authors and, if the manuscript is accepted for publication, my named report including any attachments I upload will be posted on the website along with the authors' responses. I agree for my report to be made available under an Open Access Creative Commons CC-BY license (http://creativecommons.org/licenses/by/4.0/). I understand that any comments which I do not wish to be included in my named report can be included as confidential comments to the editors, which will not be published.

I agree to the open peer review policy of the journal
