# Supplementary material for: Calcium imaging, MEA recordings, and immunostaining images dataset of neuron-astrocyte networks in culture under the effect of norepinephrine
Source: Gigascience. 2018 Dec 13;8(2):giy161. doi: 10.1093/gigascience/giy161 (PMC6351728; doi:10.1093/gigascience/giy161)
Supplement: Reviewer_2_Report_Revision_1 -- Stephen Eglen — 11/9/2018 Reviewed [file giy161_reviewer_2_report_revision_1.pdf]

Reviewer Report

Title: Calcium imaging, MEA recordings and Immunostaining images dataset of Neuron-Astrocyte Networks in Culture under the Effect of Norepinephrine

Version: Revision 1 **Date:** 11/9/2018

Reviewer name: Stephen Eglen

Reviewer Comments to Author:

Review of GIGA-D-18-00345R1

This is an exciting data set to make available, as to my knowledge simulatenous calcium imaging and MEA recordings are rare. So, first, I would thank the authors for providing the data.

The paper itself reads quite clearly, and I think I have a good understanding of what is provided. However, I have faced several problems when trying to browse the data.

line 170/171 notes a spreadsheet where all the data are described. I couldn't find it on the top-level of ftp site ftp://user76@parrot.genomics.cn nor in the accompanying http://gigadb.org/dataset/100510 page. It sounds like the spreadsheet is the main place where all the data are described, and I think this is important enough that it needs a direct link to the spreadsheet. (Apologies if I've missed it somewhere obvious.) Had I seen the spreadsheet, I would have looked for all the metadata that the paper describes.

I have a minor concern that the MEA recordings are seemingly stored in the raw .mcd files (line 80). If it were at all possible, I would like the authors to export them into hdf5 as they note can be done by the MCS data manager (line 82) as this should help determine whether they are likely to be readily used by other systems. Despite working with MEA recordings myself for nearly 20 years, I have yet to use the MCS data manager, and am wary of mcd as a suitable format for long-term storage.

I was likewise unable to get the matlab scripts (line 226) from either the URL https://github.com/gigascience/paper-bar-el2018 or from the Scripts/ folder of the ftp site. Again, have I missed something obvious? The paper does need to describe what version of Matlab (line 236) is required, as this is unclear. Also, do the scripts work only on Windows or have they been tested on Linux or Mac?

I would also appreciate some high-level summary of the dataset in the paper. Describing it as 4Tb makes it sound impressively large, but can the authors state something a bit more descriptive? (e.g. how many simultaneous recordings, how many cultures [were there 58, as line 198 suggests?] typical numbers of electrodes/ROIs per recording, number of rejected datasets]

I would be happy to see a revised version.

signed.
Stephen Eglen

Level of Interest

Please indicate how interesting you found the manuscript: Choose an item.

Quality of Written English

Please indicate the quality of language in the manuscript: Choose an item.

Declaration of Competing Interests

Please complete a declaration of competing interests, considering the following questions:

- Have you in the past five years received reimbursements, fees, funding, or salary from an organisation that may in any way gain or lose financially from the publication of this manuscript, either now or in the future?
- Do you hold any stocks or shares in an organisation that may in any way gain or lose financially from the publication of this manuscript, either now or in the future?
- Do you hold or are you currently applying for any patents relating to the content of the manuscript?

- Have you received reimbursements, fees, funding, or salary from an organization that holds or has applied for patents relating to the content of the manuscript?
- Do you have any other financial competing interests?
- Do you have any non-financial competing interests in relation to this paper?

If you can answer no to all of the above, write 'I declare that I have no competing interests' below. If your reply is yes to any, please give details below.

I declare that I have no competing interests

I agree to the open peer review policy of the journal. I understand that my name will be included on my report to the authors and, if the manuscript is accepted for publication, my named report including any attachments I upload will be posted on the website along with the authors' responses. I agree for my report to be made available under an Open Access Creative Commons CC-BY license (http://creativecommons.org/licenses/by/4.0/). I understand that any comments which I do not wish to be included in my named report can be included as confidential comments to the editors, which will not be published.

I agree to the open peer review policy of the journal
