# Supplementary material for: Calcium imaging, MEA recordings, and immunostaining images dataset of neuron-astrocyte networks in culture under the effect of norepinephrine
Source: Gigascience. 2018 Dec 13;8(2):giy161. doi: 10.1093/gigascience/giy161 (PMC6351728; doi:10.1093/gigascience/giy161)
Supplement: Reviewer_3_Report_Revision_1 -- Thomas Wachtler — 11/20/2018 Reviewed [file giy161_reviewer_3_report_revision_1.pdf]

Reviewer Report

Title: Calcium imaging, MEA recordings and Immunostaining images dataset of Neuron-Astrocyte Networks in Culture under the Effect of Norepinephrine

Version: Revision 1 **Date:** 11/20/2018

Reviewer name: Thomas Wachtler

Reviewer Comments to Author:

This dataset provides recordings from mouse cortical neuron-astrocyte cultures under electrical stimulation and pharmacological treatment with norepinephrine. Simultaneously recorded calcium imaging and electrophysiological recordings as well as immunostaining results are provided. These data can be highly valuable for functional and morphological investigations of neuron-glia networks.

The methods are well documented (protocols.io) and the manuscript describes the data in detail. However, there are inconsistencies between the description and the data, and other issues that should be addressed.

1.
- The file "readme.txt" and the folder "NegativeData" described in the manuscript are missing.
- The manuscript also mentions a "spreadsheet attached to the dataset". It is not clear which file is meant since a name is not given, but anyway I couldn't find any spreadsheet file in the repository.
- The folder "Calcium imaging and MEA analysis" is missing and only the folder "Scripts" ist present but not "Calcium GUI". The github repo (https://github.com/gigascience/paper-bar-el2018) does not exist.

2.
Many of the data directories (e.g. April2016, August2016, ...) contain no electrophysiology data, only tif files. If this is how it is intended, it should be explained. The description makes it appear as if there should be both imaging and electrophysiological data for each recording session.

3.
I did not try to verify the code provided in the Scripts directory. However, it seems like a lot of guesswork would be necessary to find out how the scripts should be used. For example, the script MEA2Raster.m loads file "18may0017.h5", but there is no such file in the dataset and it is not clear how it should be created from the files provided. A readme file that describes for example how to generate the figures from the paper would be highly useful.

4.
A general remark: Using names of months, and even weekdays, in file and folder names makes it unnecessarily hard to get a good overview of the data when browsing manually, and, more importantly, cumbersome to write scripts for systematic data analysis. I'm not asking this to be changed for the

current dataset, but would like to suggest that in the future the authors consider using standard datetime notation.

Level of Interest

Please indicate how interesting you found the manuscript: Choose an item.

Quality of Written English

Please indicate the quality of language in the manuscript: Choose an item.

Declaration of Competing Interests

Please complete a declaration of competing interests, considering the following questions:

- Have you in the past five years received reimbursements, fees, funding, or salary from an organisation that may in any way gain or lose financially from the publication of this manuscript, either now or in the future?
- Do you hold any stocks or shares in an organisation that may in any way gain or lose financially from the publication of this manuscript, either now or in the future?
- Do you hold or are you currently applying for any patents relating to the content of the manuscript?
- Have you received reimbursements, fees, funding, or salary from an organization that holds or has applied for patents relating to the content of the manuscript?
- Do you have any other financial competing interests?
- Do you have any non-financial competing interests in relation to this paper?

If you can answer no to all of the above, write 'I declare that I have no competing interests' below. If your reply is yes to any, please give details below.

I declare that I have no competing interests

I agree to the open peer review policy of the journal. I understand that my name will be included on my report to the authors and, if the manuscript is accepted for publication, my named report including any attachments I upload will be posted on the website along with the authors' responses. I agree for my report to be made available under an Open Access Creative Commons CC-BY license (http://creativecommons.org/licenses/by/4.0/). I understand that any comments which I do not wish to be included in my named report can be included as confidential comments to the editors, which will not be published.

I agree to the open peer review policy of the journal
